# Efficacy of Cognitive Rehabilitation Therapy on Poststroke Depression among Survivors of First Stroke Attack in Ibadan, Nigeria

**DOI:** 10.1155/2017/4058124

**Published:** 2017-06-27

**Authors:** Olugbemi Olukolade, Helen O. Osinowo

**Affiliations:** ^1^Family Medicine Department, University College Hospital, Ibadan, Oyo, Nigeria; ^2^Department of Psychology, University of Ibadan, Ibadan, Oyo, Nigeria

## Abstract

**Background and Purpose:**

Poststroke depression (PSD) is a common complication after stroke. There is no adequate treatment for PSD. This study examined efficacy of cognitive rehabilitation therapy (CRT) in the treatment of PSD among stroke survivors.

**Methods:**

An experimental design, 30 participants with poststroke depression were randomly assigned into 3 groups of cognitive rehabilitation therapy (CRT), psychoeducation (PE), and the control group (CG). CRT consisted of nine sessions with three-phased sessions focusing on activity stimulation, negative thoughts, and people contacts, PE consisted of nine sessions focusing on knowledge on stroke and poststroke depression, and the CG group was on the waiting list. The BDI scale was used for assessing PSD at posttest.

**Results:**

There was a significant difference in the efficacy of CRT, PE, and the CG on PSD, with CRT–CG mean difference of −9.4 ± 3.11 and PE–CG 1.0 ± 3.83. Furthermore, stress was not a confounding variable on the efficacy of CRT. The type of therapy significantly influenced PSD at posttest, with the CRT having greater mean reduction to CG (−11.1 ± 3.1) than PE to the CG (3.0 ± 3.8).

**Conclusions:**

Cognitive rehabilitation therapy significantly reduced poststroke depression. Hence, it should be integrated as an adjunct treatment of poststroke depression.

## 1. Introduction

The global burden of disease is on the increase, and up to 12% of this was attributed to mental disorders [1, 2]. Recently, it has been estimated that mental illness will account for about 15% of this rising number in the future [2]. Among the commonest mental disorders, depression is the second most important cause of disability [3–6].

Poststroke depression (PSD) is not only a common sequel after stroke but also one of the most frequent complications of stroke, with a prevalence ranging between 20 and 60% [7]. Poststroke depression has the same signs and symptoms as does major depressive disorder (MDD), (including disturbed sleep, a lack of interests, guilt or a preoccupation of thought, reduced energy, diminished concentration ability, disturbed appetite, psychomotor agitation or retardation, and thoughts of death or suicide). Four or more of these symptoms, in the presence of depressed mood or anhedonia (the loss of pleasure), for a duration of 2 weeks or longer will satisfy criteria for poststroke depression.

Poststroke depression adversely affects a patient's chance (and rate) of recovery, particularly those with major depressive disorder who have been found to be less compliant with rehabilitation, more irritable, and demanding and may experience personality change (www.caring.com 2016). Thus, it has been associated with more than a 3-fold increase in mortality rates for as long as 10 years after stroke [8] especially due to the many side effects of medications to treat depression which many times make the team overlook the disorder despite its negative impact.

The association between depression and stroke has long been contemplated for its negative impact on an individual's rehabilitation, family relationships, and quality of life [9].

It is therefore appropriate to have the diagnosis and treatment of depression after a stroke illness to have substantial benefits to the patient through improved medical status, enhanced quality of life, a reduction in the degree of pain and disability, and improved treatment compliance and cooperation. Appropriate diagnosis and treatment of depression can shorten the rehabilitation process and lead to more rapid recovery and resumption of routine. It can also save health care costs (e.g., eliminate nursing home expenses) and long-term adjunct treatments.

Despite the high prevalence of depression as a psychopathology among stroke survivors, only one-third of moderately to severely depressed patients in most samples were referred for treatment by mental health professionals during their hospital stay [10]. This low treatment rate, which is consistent with that of other studies, indicates that poststroke pathology most especially depression remains largely untreated. This situation may be especially problematic for the subset of patients who also report hopelessness in the range of potential suicide risk. Many times, neurologists have significant restraint in using psychiatric medication because of comorbid interactions and side effects caused by this poststroke depression such as lowered adherence, lower quality of life, lowered sexual interest with impact on the survivor and the partner(s), and many more. To this end, determining the effectiveness of cognitive rehabilitation therapy (CRT) in the treatment of poststroke depression is important if we are to gain better understanding to the alternative, cost effective treatment in stroke populations.

Cognitive rehabilitation is a systematically applied set of therapeutic services designed to improve cognitive functioning and participation in activities that may be affected by difficulties in one or more cognitive domains. Cognitive rehabilitation therapy is a nonmedicinal treatment that involves individually tailored cognitive exercises developed by a neuropsychologist to retrain and/or improve cognition through correction of neurocognitive deficits such as attention; visual, spatial memory; and also depressive symptoms.

Cognitive rehabilitation therapy (CRT) is an effective treatment of depression in the stroke survivor population [11] and in the elderly [12]. It is evident that the rehabilitation procedure for this epidemic is confronted by many hindrances worldwide. Cognitive rehabilitation therapy has been proven to be as effective as medication in controlled trials, approximately 50% of patients with depression experience clinically meaningful improvement [13]. However, within the cerebrovascular accident victims worldwide and more especially in Nigeria, the use of these psychological therapies such as cognitive rehabilitation therapy which combines these approaches remains largely elusive [14].

Evidence-based reviews and meta-analyses have shown that CRT can rival the effects of certain medications in the treatment of PSD and it includes the use of cognitive behavior therapy (CBT), problem solving, and psychoeducation; that CRT can enhance the effectiveness of medication alone (the US Heart Foundation rated the combination as grade B level but more recently published studies suggest that the higher grade A level may now be warranted); and that CRT effects are “real” and can impact pain pathways deep within the brain as judged from laboratory studies when effectiveness of components of CRT have been examined.

## 2. Methods

### 2.1. Design

This is a randomized clinical trial using an experimental design. The design was appropriate to assess the therapeutic efficacy of the treatment modality of cognitive rehabilitation therapy on poststroke depression as against psychoeducational training and waiting list control which was in three homogenous groups, namely, homogenous except treatment modality.

A baseline score was obtained at entry for participants; then in the interventions, it was introduced to groups A (CRT) and B (PET) of the three groups, that is, group A (cognitive rehabilitation therapy), group B (psychoeducation), and group C (usual care). Assessments were done at first contact, the third session, the sixth session, and at the end of the ninth session (while the interventions were usually held weekly all over a period of 3 and a half months).

The experimental groups received a pretest, the intervention, and posttest on the dependent variable (poststroke depression) using the independent variable (CRT).On other hand, the control group received only pretest and posttest only (though the control also received weekly text messages over the phone on general greetings and quotable quotes). Finally, the follow-up was collected on both the experimental and control groups using the dependent variable a week after the intervention was done, preassessment and postassessment.

The design can be represented in Table 1.

Those who scored ≥11 on the Beck Depression Inventory are offered the opportunity to consent for the intervention study, with diagnosis of depression validated by a diagnostic interview using DSM IV criteria.

The cognitive rehabilitation therapy (CRT) consisted of nine sessions with first three sessions focusing on activity stimulation, second three focusing on negative thoughts, and the third focusing on people contacts; psychoeducation therapy (PE) consisted of nine sessions focusing on knowledge on stroke and poststroke depression; and the control group (CG) (usual care) on waiting list focuses on the basic care often provided for the stroke survivors in the hospital including neurological care physiotherapy devoid of any form of psychotherapeutic interventions.

## 3. Setting

The study was carried out in the tertiary health care centre in University College Hospital (UCH), Ibadan. The hospital is a major tertiary hospital in Nigeria with a reputation of being the first teaching hospital, and it has a wide array of facilities. University College Hospital, Ibadan, is a 1000-bedded tertiary hospital which serves as a referral centre from primary and secondary health centres all over Nigeria.

### 3.1. Sampling Technique

The patients were purposefully drawn from the stroke survivor's population. The patients were assessed on BDI, and only those who score more than 11 were recruited to be part of the study. They were subsequently randomly assigned using a table of random numbers into the three groups of cognitive rehabilitation therapy (CRT), psychoeducation therapy (PET), and the control group (CG).The sample size for the study was calculated using Lagos registry prevalence of stroke of 114 in 100,000 [15]. Therefore, the sample size for stroke patients for the study was 89.

However, 90 patients were used for the first phase study, while this intervention stage had a double blind selection of 30 stroke survivors of 10 participants in each of the three groups.

### 3.2. Participants

A total of 35 first ever stroke survivors were eligible participants for the study, although 30 participated and were randomly assigned into the groups, 13 males (43.3%) and 17 females (56.7%). The gender and educational qualification of the participants were controlled by randomly assigning them into the three groups specifically using the level of education (using the fish bowl method) with the CRT group (5 males, 5 females), PE group (4 males, 6 females), and CT (4 males, 6 males).

Graphical representation of the groups' educational qualification after randomization is presented in Table 2.

### 3.3. Research Instruments

#### 3.3.1. The Beck Depression Inventory

This is a 21-item scale that measures clinical depression. The original version of the Beck Depression Inventory was published in 1961 and subsequently revised [16–19]. It had been extensively used especially in assessing and monitoring changes with cognitive therapy, and it was used to measure the level of poststroke depression. The long form of 21 items was used to provide a quantitative assessment of the severity of depression. <10 represents minimum or no depression; 10–18 indicates mild to moderate; 19–29 shows moderate to severe depression; and 30–63 shows severe depression. Reliability studies showed a test-retest correlations having ranged from 0.48 to 0.90 [19]. It had been extensively used in Nigeria with various validations; in the present study, it had a Cronbach alpha coefficient of 0.77 in the stroke population.

#### 3.3.2. Life Event Stress Scale

This is a 42-item scale that measures stressful life events. Life events were assessed (i.e., undesirable and severe). These events were also classified as primarily interpersonal (e.g., death of a loved one) or related to achievement (e.g., loss of employment). The patients were asked about events that occurred during the 6-month period before the onset of the current poststroke-depressive episode. Also, the comparison subjects were asked about events during the 6-month period immediately preceding the interview. The 6-month time frame was used because it has been shown to be the optimal period for detecting the significant effect of life events on subsequent depressive onset, and other studies have used this standard; it had a 0.84 Cronbach alpha reliability score.

### 3.4. Procedure

Request for consent was taken from the relevant authorities and the participants before being recruited into the study. The ethical board of the UI/UCH Research Committee approved the study in its entirety. The UI/UCH Ethics Committee assigned number is UI/EC/11/0296.

#### 3.4.1. Exclusion/Inclusion Criteria

Exclusion criteria are the following:

- If they had a major psychiatric disorder other than effective disorders (e.g., schizophrenia or a current psychotic episode)
- If they had reported a depressive episode in the weeks before the time of the stroke/myocardial infarction
- If they had a comorbid intracerebral disease
- If the clinician judged that they were unable to understand the informed consent procedure (e.g., because of severe aphasia or dementia) after having administered the minimental state examination (MMSE) and the Barthel Index
- If the patient level of exposure to education cannot sustain executive function discussion.

Inclusion criteria are the following:

- Patient must be having first ever stroke.
- If participants were given written informed consent, this was approved by the UI/UCH medical ethics committee and accepts to be in the study.
- The respondents had fulfilled first part of the study measuring physical disability, perceived social support, health-related quality of life, and depression inventory scale.

None of the participants were on any antidepressant/psychotropic medication for the treatment of any form of mood disorders in the past/six months or at the time of the collection of data or intervention.

The responses were later looked into to check those who had fulfilled the criterion of the study to be included. All three stages of pretreatment assessment, psychotherapeutic intervention, and posttreatment assessment were carried out for the cognitive rehabilitation therapy and psychoeducation, while the no-treatment group had no psychotherapeutic intervention but postassessment.

## 4. Results

**Figure 1:**
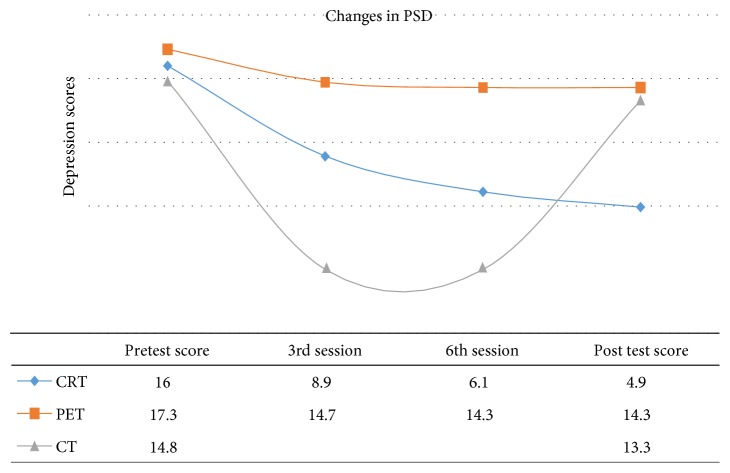
The different levels of depression scores at intervals.

The effect of cognitive rehabilitation therapy (CRT) on poststroke depression in the study was from a score of 16 to 4.9 while that of the control group (CG) was from14.8 to 13.3 and that of the psychoeducation group (PG) was from 14.8 to 13.3; data are shown in Figure 1. This number implies that there was a significant reduction from the level of poststroke depression among the CRT group than that of the PE group or CG.

At pretest, the different groups of the study, namely, CRT (cognitive rehabilitation therapy), PET (psychoeducation therapy), and the CT (waiting list control therapy), had a pretest group mean score CRT = 16, PET = 17.3, and CT = 14.8, respectively. By the second session, the mean score for the CRT was almost half while the PET marginally reduced to 14.7, and at the 6th session of about 2 months plus, the mean score was less. There was no significant depression score on the BDI for the CRT group with a score of 6.1 while the PET was stable, and while at posttest, CRT was 4.9, PET 14.3, and control 13.3 showing the curve of treatment efficacy for CRT.

The first hypothesis states that stroke survivors who are exposed to cognitive rehabilitation therapy (CRT) will have significant reduction in their poststroke depression at posttest than at pretest than the waiting list control (CT) survivors who were tested with the independent *t*-test statistics, and the result is presented on Table 3.

Table 3 shows that the type of therapy has significant influence on poststroke depression pretest and posttest (*t* = −8.107; df = 18; *p* < 0.001). This significant difference can be observed in the mean where stroke survivors in the CRT had a greater reduction ($\bar{X}=-11.10$) in the level of poststroke depression than stroke survivors in the waiting list ($\bar{X}=-1.50$) with a mean difference of −12.600. Examination of the result reveals that stroke survivors who were treated with the CRT reported greater reduction in the level of poststroke depression pretest and posttest than the stroke survivors who were in the waiting list (control). The hypothesis was therefore confirmed.

The second hypothesis states that stroke survivors who are exposed to cognitive rehabilitation therapy will have significant positive difference to the psychoeducation therapy and waiting list control on their level of poststroke depression controlling for stressful events. This hypothesis was analyzed using analysis of covariance (ANCOVA), and the result is presented in Table 4.

Table 5 reveals significant variation among the levels of psychotherapy on poststroke depression [*F* (2, 27) = 8.639; *p* < .01]. This implies the different levels of psychotherapy were significantly different on poststroke depression; hence, a post hoc analysis was carried out to find out where the difference is. The result of the post hoc analysis is presented in Table 6.

Table 6 shows the multiple comparison tables of the mean scores obtained by the different levels of psychotherapy. Examination of the table reveals that the difference in the mean score between CRT and PET (−9.40, *p* < .05) was significant and the difference in the mean score between CRT and CT (−8.40, *p* < .05) was significant (see Table 6). However, the difference in the mean score between PET and CT (1.00, *p* > .05) was not significant.

Table 7 shows the level of significance of levels of psychotherapy when stressful event is introduced as a covariate to the levels of psychotherapy. The result shows that before the introduction of the covariate, the value of levels of psychotherapy as seen in Table 5 was [*F* (2, 27) = 8.639; *p* < .01] and, when the covariate was introduced, the value of levels of therapy on poststroke depression was [*F* (2, 27) = 7.361; *p* < .01]. This implies that the introduction of stressful events had no significant effect on the level of psychotherapy; hence, stressful events did not covary with levels of psychotherapy to have an effect on poststroke depression.

## 5. Discussion

The study has shown that stroke survivors who were exposed to cognitive rehabilitation therapy, psychoeducation therapy, and waiting list control differed significantly on their level of poststroke depression controlling for stressful events. It was shown that there are differences in the poststroke depression of the different treatment modalities after therapy and that the introduction of stressful life events does not change nor influence this result. This is similar to the result of Cicerone et al. [20] in which there was a comparison of cognitive rehabilitation therapy (CRT) to other forms of therapy to assess relative to comparative effectiveness and it was found that greater improvement was in the standard cognitive rehabilitation therapy (CRT) group for the management of symptoms at follow-up. Findings by Tiersky et al. [21] when they tested the efficacy of a comprehensive rehabilitation therapy program with cognitive rehabilitation sessions found that the treatment group improved significantly in depression compared to the control at 1-month and 3-month follow-ups.

In greater detail, stroke survivors who are exposed to cognitive rehabilitation therapy (CRT) had significant reduction in their poststroke depression at posttest than at pretest than the waiting list control (CT) survivors in the level of poststroke depression among the cognitive rehabilitation group than the waiting control list group. These results are consistent with the findings of Sarajuuri et al. [22] which evaluated survivors, following a comprehensive 6-week neurorehabilitation program with psychotherapy against a waiting list of conventional care, and found that even at two-year follow-up, the treatment group improved significantly more than the control group. Consistent with this also is the Braunling-Mcmorrow et al. study [23] on the effect of multifaceted rehabilitation services on functional outcomes for patients; they found in the study that using the rehabilitation treatment model for neuropsychologically impaired participants had significant treatment gains of approximately 1.5 levels.

Furthermore, a study by Rattok et al. [24] assessed three groups with varying treatment combinations of cognitive rehabilitation therapy (CRT), personal counseling/information, and small group exercises with each group receiving 400 hours each, and it was discovered that all the three groups mix well with effectiveness but some superior results especially in the intra- and interpersonal functions of the cognitive rehabilitation therapy (CRT) group were more emphasized. Finally, Macdonald et al. [25] tested the efficacy of remediation, cognitive rehabilitation therapy (CRT), and waiting list control with the focus of their study on the mood disturbances (depression and anxiety), and they observed that the skill training group (CRT) did improve differentially on their outcome measure variable; however, there was no difference between the other primary and secondary outcomes in the study. It is of major concern that none of the depressed patients in this study was on any antidepressant treatment or referred for psychotherapy prior to the study itself. This is despite of the well-documented literature on the increased disability, worse rehabilitation outcome, and higher mortality in depressed survivors [26, 27].

The psychoeducation group in the intervention phase had significantly higher poststroke depression. This is possibly due to the fact that the increased education on the risks, course, and causes of stroke is likely to make sufferers to be more aware of potential recurrence, probable length of illness which heightens distress.

This study has established the efficacy of cognitive rehabilitation therapy in the treatment of poststroke depression and the impact of perceived social support on the development and progression of poststroke depression. Cognitive rehabilitation therapy differs significantly with other psychological forms of treatment such as psychoeducation and the waiting list control in rehabilitation results. The study has a small sample size among which was due to the individualizing of the sessions because of possible lower effect of group therapy. This may have implications on the generalizations of the effectiveness of CRT as seen in the study. Further, future studies would be needed to address this and to replicate the result found in this study.

Depression following stroke is real even in African societies and is a widespread phenomenon that must be recognized and managed by health care professionals and rehabilitators. Some form of treatment must be instituted as soon as the disorder presents itself if we are to effectively and humanely care for this at risk population.

## Figures and Tables

**Table 1:** 

Quasi-experimental groups (QEG)	Pretest assessment (PreTA)	Treatment types	Posttest assessment
QEG1	PreTA1	CRT	PostTA1
QEG2	PreTA2	PET	PostTA2
QEG3	PreTA3	Control (usual care)	PostTA3

CRT: cognitive rehabilitation treatment; PET: psychoeducation treatment; control: usual treatment without psychological-based intervention.

**Table 2:** 

Level of education in each group	Total number	Number in CRT group	Number in PE group	Number in CT
No formal certificate	5	1	2	2
Pri cert only	8	3	3	2
Sec cert only	5	2	1	2
Tertiary only	9	3	3	3
Doctoral or more	3	1	1	1

**Table 3:** Summary of independent *t*-test comparing CRT and waiting list control on poststroke depression.

	Therapy						
	Type	*N*	$\bar{X}$	SD	df	*t*	*p*
Poststress depression	CRT	10	−11.10	3.107	18	−8.107	<.001
	CT	10	1.50	3.808			

**Table 4:** A descriptive table of different levels of psychotherapy.

Psychotherapy	*N*	Mean	Std. deviation
CRT	10	4.90	2.07
PET	10	14.30	7.64
CT	10	13.30	5.45

**Table 5:** Summary of one-way analysis of variance comparing different levels of psychotherapy on poststroke depression.

Source of variation	SS	df	MS	*F*	*p*
Between	533.06	2	266.53	8.639	<.01
Within	833.10	27	30.85		
Total	1366.16	29			

**Table 6:** Summary of post hoc comparison in mean of the different levels of psychotherapy.

Therapy	Therapy	Mean	Mean difference	Sig
CRT	PET	14.30	−9.40	.001
	CT	13.30	−8.40	.002
PET	CRT	4.90	9.40	.001
	CT	13.30	1.00	.690
CT	CRT	4.90	8.40	.002
	PET	14.30	−1.00	.690

**Table 7:** Summary of analysis of covariance (ANCOVA) when a stressful event is introduced as a covariate in the effect of levels of psychotherapy on poststroke depression.

Source of variation	SS	df	MS	*F*	*p*
Stress	2.68	1	2.68	0.084	>.05
Therapy	470.21	2	235.108	7.361	<.01
Total	1366.16	29			
